# Gold-Dispersed Copper Ferrite Magnetic Hydrogel: A Potent Antibacterial Nanozyme Platform

**DOI:** 10.3390/gels12090760

**Published:** 2026-08-25

**Authors:** Ankush Sheoran, Ekta Arya, Meha Bhargava

**Affiliations:** 1 State Key Laboratory of Flexible Electronics (LoFE) & Institute of Advanced Materials (IAM), School of Flexible Electronics (Future Technologies), Nanjing Tech University, Nanjing 211816, China; 2 Department of Physics, Guru Jambheshwar University of Science and Technology, Hisar 125001, Haryana, India

**Keywords:** nanozyme, copper ferrite, gold nanoparticles, magnetically retrievable hydrogel, POD-like activity, ROS, antibacterial

## Abstract

A gold-dispersed CuFe_2_O_4_/alginate magnetic hydrogel was developed as a nanozyme-based antibacterial platform. CuFe_2_O_4_ nanoparticles were hydrothermally synthesized and incorporated into an alginate hydrogel with in situ-generated Au nanoparticles. ICP-OES analysis confirmed the successful incorporation of Fe (7.16%), Cu (3.63%), and Au (0.38%), with an Fe–Cu ratio consistent with CuFe_2_O_4_. The hydrogel exhibited ferromagnetic behaviour (M_s_ = 12.35 emu g^−1^) and potent POD-like activity, generating ROS with high catalytic efficiency (K_m_ = 0.01 mM; V_max_ = 13.47 × 10^−8^ M s^−1^). The hydrogel also showed significant GSH-depleting ability and could be reused for up to six cycles. Antibacterial assays demonstrated enhanced killing of *S. aureus* and *E. coli* when combined with H_2_O_2_ at reduced concentrations. Cytocompatibility studies demonstrated favourable in vitro cytocompatibility, with cell viability remaining above 85%, and the hydrogel exhibited good swelling and mechanical properties. With its magnetic responsiveness, favourable in vitro cytocompatibility, and enhanced antibacterial activity, this nanozyme-integrated hydrogel represents a promising platform for further investigation in antibacterial wound-dressing applications.

## 1. Introduction

The ever-rising threat of antimicrobial resistance (AMR) has emerged as one of the most critical public health crises of the 21st century, with the World Health Organization identifying it as a top ten global health threat [1]. The proliferation of drug-resistant bacteria, particularly multidrug-resistant (MDR) strains such as methicillin-resistant *Staphylococcus aureus* (MRSA) and carbapenem-resistant Enterobacteriaceae, has rendered many conventional antibiotics increasingly ineffective, leading to prolonged hospitalizations, higher medical costs, and a considerable increase in mortality [2]. Among the most challenging consequences of this crisis are chronic infections, which are notoriously difficult to eradicate due to the formation of protective biofilms, the ability of bacteria to enter a metabolically dormant state, and the harsh, inflammatory microenvironment that they create within host tissues [3,4,5]. To counter these persistent infections, numerous therapeutic strategies have been developed over the years, ranging from the synthesis of novel synthetic antibiotics and antimicrobial peptides to the use of bacteriophages, anti-biofilm enzymes, and alternative physical methods like photothermal and photodynamic therapies [6,7,8]. Despite these advances, the clinical translation and long-term efficacy of many of these approaches are hampered by issues such as rapid development of secondary resistance, poor bioavailability, systemic toxicity, high production costs, and inadequate penetration or activity within the complex infection microenvironment [9,10,11]. As a result, there remains a pressing need for the exploration of novel, broad-spectrum antimicrobial agents that operate *via* non-specific mechanisms, thereby bypassing traditional resistance pathways, while offering high stability, low toxicity, and the capacity to function effectively in complex biological settings [12].

In recent years, nanozyme-based strategies have emerged as a compelling alternative to conventional antimicrobial agents, offering the unique advantage of mimicking natural enzymatic activities while overcoming the inherent instability and high production costs of natural enzymes [13,14,15]. Among various nanozyme platforms, metal oxide nanoparticles, particularly spinel ferrites (MFe_2_O_4_, where M represents a divalent cation), have garnered significant attention due to their exceptional chemical stability, tuneable catalytic performance, intrinsic magnetic properties, and excellent biocompatibility [16,17,18]. Copper ferrite (CuFe_2_O_4_) is of particular interest owing to its peroxidase-like (POD) and glutathione-depleting (GSH) activities, which enable the generation of reactive oxygen species (ROS) for effective bacterial eradication [19,20,21]. Furthermore, the incorporation of noble metals such as gold (Au) into spinel ferrite structures is known to enhance catalytic efficiency through favourable electronic interactions, improved electron transfer, and increased active-site exposure, thereby amplifying their antibacterial performance [22,23,24]. However, despite these promising attributes, the practical application of nanozyme powders is often constrained by issues related to poor dispersibility, rapid aggregation, limited retention at target sites, and potential cytotoxicity upon direct administration [25,26].

To overcome these limitations, the integration of nanozymes into hydrogel matrices has emerged as a highly promising approach for biomedical applications. Hydrogels have become one of the most versatile biomaterial platforms for wound healing owing to their high water content, excellent biocompatibility, injectability, and ability to mimic the native extracellular matrix [27,28,29]. Nanozyme-incorporated hydrogels have attracted considerable attention because they combine the structural advantages of hydrogels with the catalytic activities of nanomaterials [30,31]. Through enzyme-mimicking functions, nanozymes can scavenge excessive ROS, alleviate oxidative stress, suppress inflammation, and create a favourable microenvironment for tissue repair [32,33,34]. Consequently, various nanozyme hydrogels have been developed for biomedical applications, including antibacterial therapy, tissue regeneration, biosensing, cancer treatment, and diabetic wound healing [35,36]. Despite these advances, many nanozyme hydrogels primarily rely on ROS regulation and antibacterial activity, while exhibiting limited capability to actively stimulate angiogenesis, cellular proliferation, and tissue remodelling [37,38]. Moreover, insufficient catalytic efficiency, poor long-term bioactivity, and inadequate multifunctionality continue to restrict their therapeutic performance, highlighting the need for more effective nanozyme-based wound-healing systems [39].

Sodium alginate, a naturally derived polysaccharide, has been widely explored as a hydrogel matrix due to its excellent biocompatibility, ease of gelation in the presence of divalent cations, and ability to provide a moist wound environment conducive to healing [40,41,42]. The incorporation of functional nanomaterials into alginate hydrogels has been demonstrated to impart additional therapeutic properties while maintaining the favourable physicochemical characteristics of the hydrogel scaffold [43,44,45]. In this context, the development of an alginate-based hydrogel incorporating gold-dispersed copper ferrite (Au-CuFe_2_O_4_) nanozymes represents a rational strategy to achieve enhanced antibacterial activity. The incorporation of CuFe_2_O_4_ provides potent POD and GSH-mimicking activities for ROS generation, while Au doping further amplifies the catalytic efficiency through improved electron transfer kinetics and increased ROS generation. Furthermore, the incorporation of nanozymes within an alginate hydrogel matrix can facilitate localized retention and handling while potentially reducing some limitations associated with free nanozyme administration [46].

In this study, we synthesized CuFe_2_O_4_ magnetic nanoparticles and subsequently incorporated them into a sodium alginate hydrogel matrix, with gold being added in situ during the hydrogel formation to achieve a gold-dispersed magnetic hydrogel (Au-CuFe_2_O_4_ hydrogel). The POD and GSH activities of the hydrogel were systematically evaluated to assess its catalytic performance. Furthermore, the antibacterial efficacy of the Au-CuFe_2_O_4_ hydrogel was investigated against representative Gram-positive and Gram-negative bacterial strains. The findings of this work demonstrate the potential of this nanozyme-integrated hydrogel as an effective antimicrobial platform, providing a promising basis for further investigation in antibacterial wound-dressing applications.

## 2. Results and Discussion

### 2.1. Synthesis

**Figure 1:**
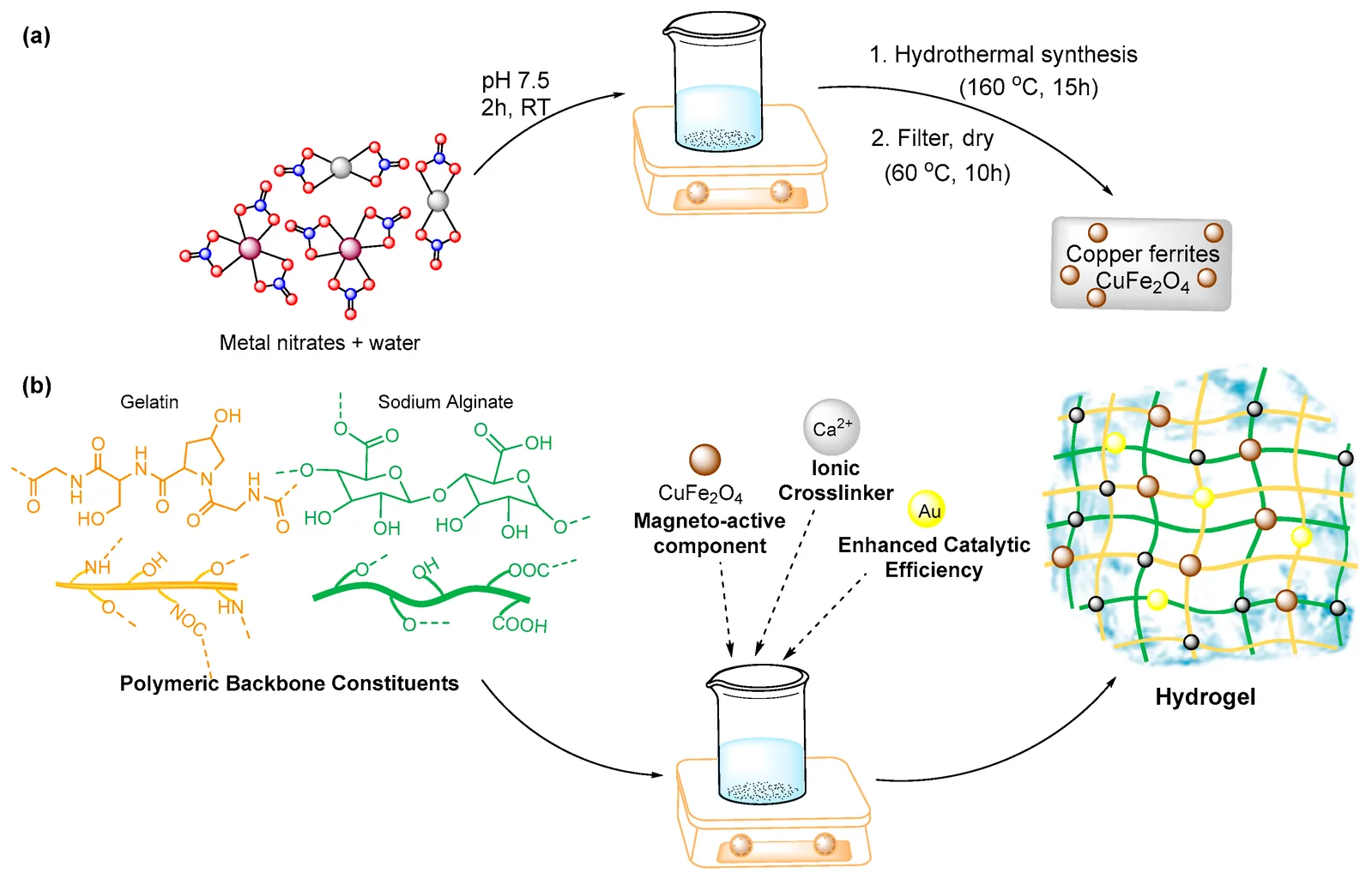
Schematic representation of the (**a**) synthesis of CuFe_2_O_4_ NPs and (**b**) Au-CuFe_2_O_4_ hydrogel.

The CuFe_2_O_4_ nanoparticles were prepared using a hydrothermal approach and subsequently dispersed in a sodium alginate solution containing gelatine as a stabilizer. The mixture was crosslinked with CaCl_2_ to form the hydrogel, followed by in situ reduction of HAuCl_4_ with ascorbic acid to generate gold nanoparticles within the hydrogel matrix, resulting in the final Au-CuFe_2_O_4_ hydrogel (Figure 1).

### 2.2. Characterization of the Synthesized Materials

**Figure 2:**
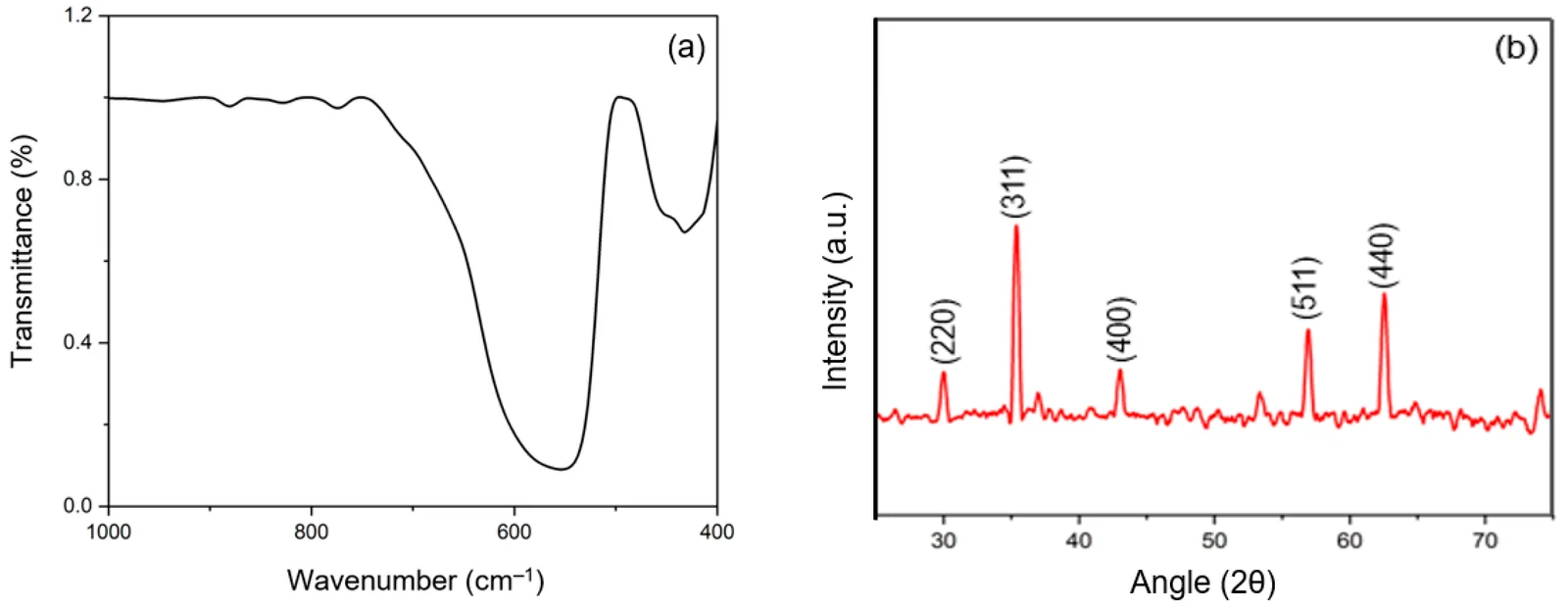
(**a**) FT-IR spectrum and (**b**) PXRD analysis of CuFe_2_O_4_ NPs.

FT-IR Spectra: The FT-IR spectrum for the synthesized CuFe_2_O_4_ nanoferrites is shown in Figure 2a. It is well reported that the FT-IR spectra of ferrites exhibit two absorption bands corresponding to M–O bond stretching vibrations in tetrahedral and octahedral regions [47]. The absorption band observed in the range of 500–600 cm^−1^ corresponds to the M–O stretching vibrations of tetrahedral clusters for all of the nanoferrites. However, the band corresponding to the M–O stretching vibrations of octahedral clusters could be observed around 400 cm^−1^ in the case of octahedral M–O stretching vibrations.

PXRD Analysis: A powder X-ray diffraction study was carried out to analyse the formation of various phases and to explore the purity of the synthesized spinel nanoferrite (Figure 2b). The crystallite size of ferrite composition was calculated from the line broadening of the most intense peak (311) by using the classical Scherrer equation [48]:

(1) D_hkl_ = 0.9λ/βcos θ

where D_hkl_ is the crystallite size, λ is the wavelength of the radiation used, β is the full width at half-maximum, and θ is the angle of diffraction.

The crystallite size of the prepared sample was found to be 26 nm (Table 1). The lattice parameter for copper ferrites was calculated using the Le Bail refinement method (built in TOPAS V2.1 of BRUKER AXS), and the value was observed to be 8.42 Å.

**Figure 3:**
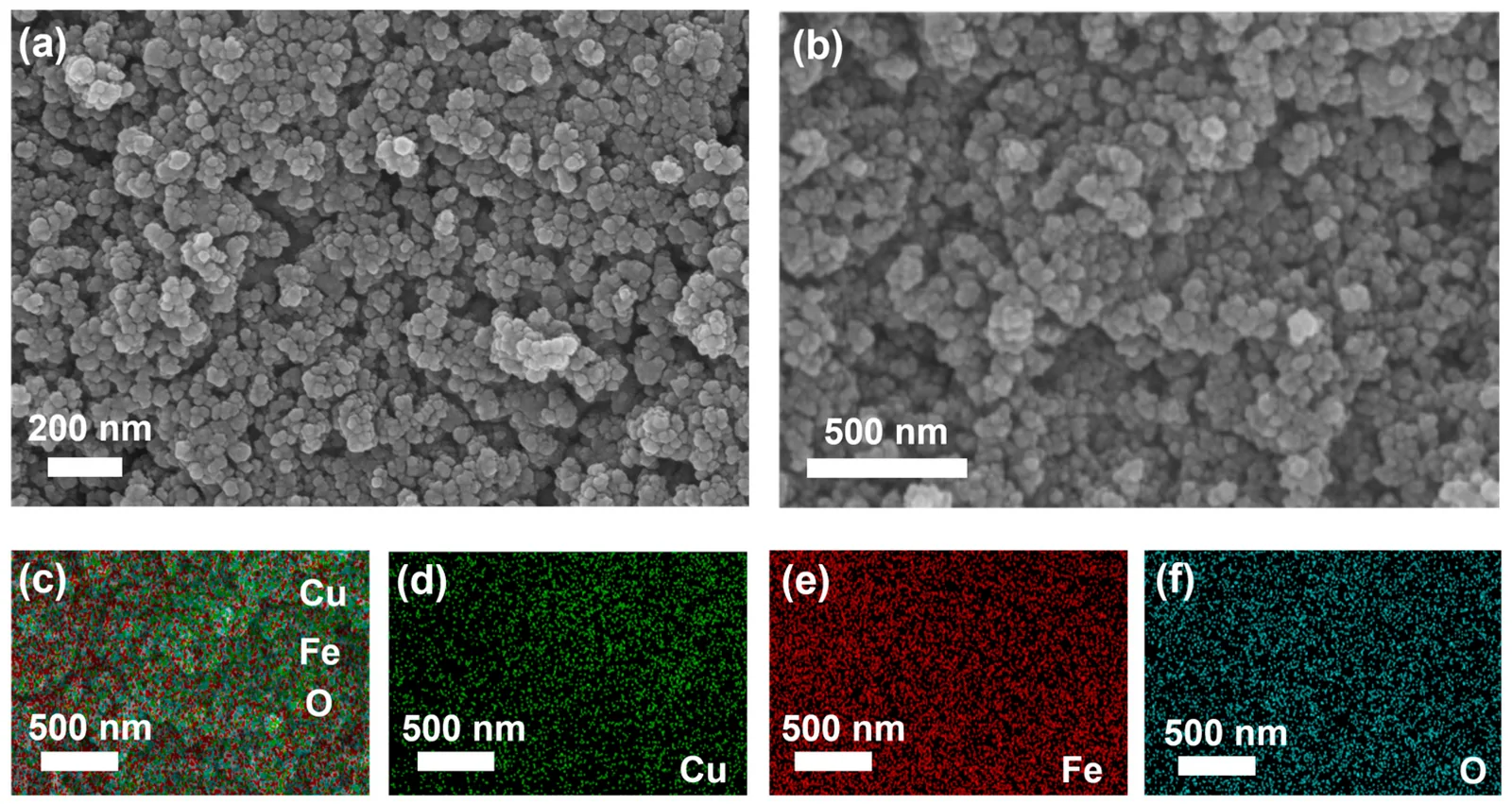
(**a**) SEM images, (**b**–**f**) elemental mapping of CuFe_2_O_4_ NPs.

Structural Analysis of CuFe_2_O_4_ NPs: The analysis of the shape and size of the synthesized nanoferrite was investigated by scanning electron microscopy (SEM) and electron-dispersive spectroscopy (EDS) techniques. Typical SEM images of the synthesized CuFe_2_O_4_ NPs are shown in Figure 3a. The images indicate a homogeneous nature of nanoferrite with small grain size distribution. The sample exhibited a quasi-spherical shape, with an average grain size of 15–25 nm. The Figure 3b represents the selected area for elemental mapping. The elemental mapping of CuFe_2_O_4_ nanoparticles synthesized by the hydrothermal route is shown in Figure 3c–f. The results confirm the uniform distribution of all of the constituent elements present in the synthesized sample.

Structural Analysis of Hydrogel: The surface morphology of the Au-CuFe_2_O_4_/alginate hydrogel was examined using scanning electron microscopy (SEM) and transmission electron microscopy (TEM).

**Figure 4:**
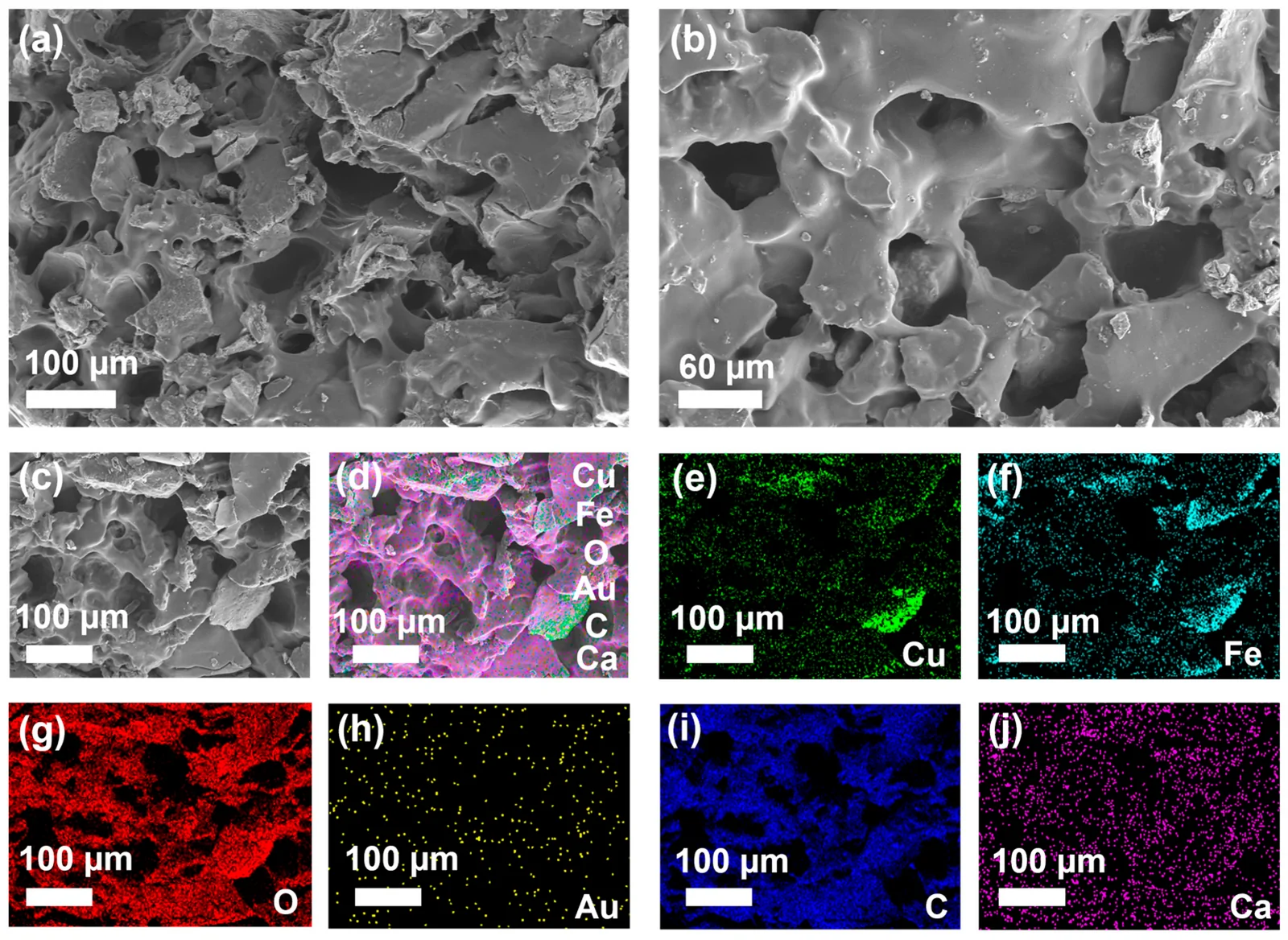
(**a**,**b**) SEM images and (**c**–**j**) elemental mapping of synthesized hydrogel.

The SEM images revealed a highly porous, interconnected, three-dimensional network structure, which is characteristic of alginate-based hydrogels formed *via* ionic crosslinking (Figure 4a,b). The porous architecture is advantageous for biomedical applications, as it facilitates nutrient diffusion, oxygen exchange, and cell infiltration. Additionally, the incorporation of CuFe_2_O_4_ nanoparticles and in situ-generated Au nanoparticles within the hydrogel matrix appeared uniformly distributed, with no significant aggregation observed, indicating successful dispersion and stabilization of the nanozymes within the polymer network (Figure 4c–j).

**Figure 5:**
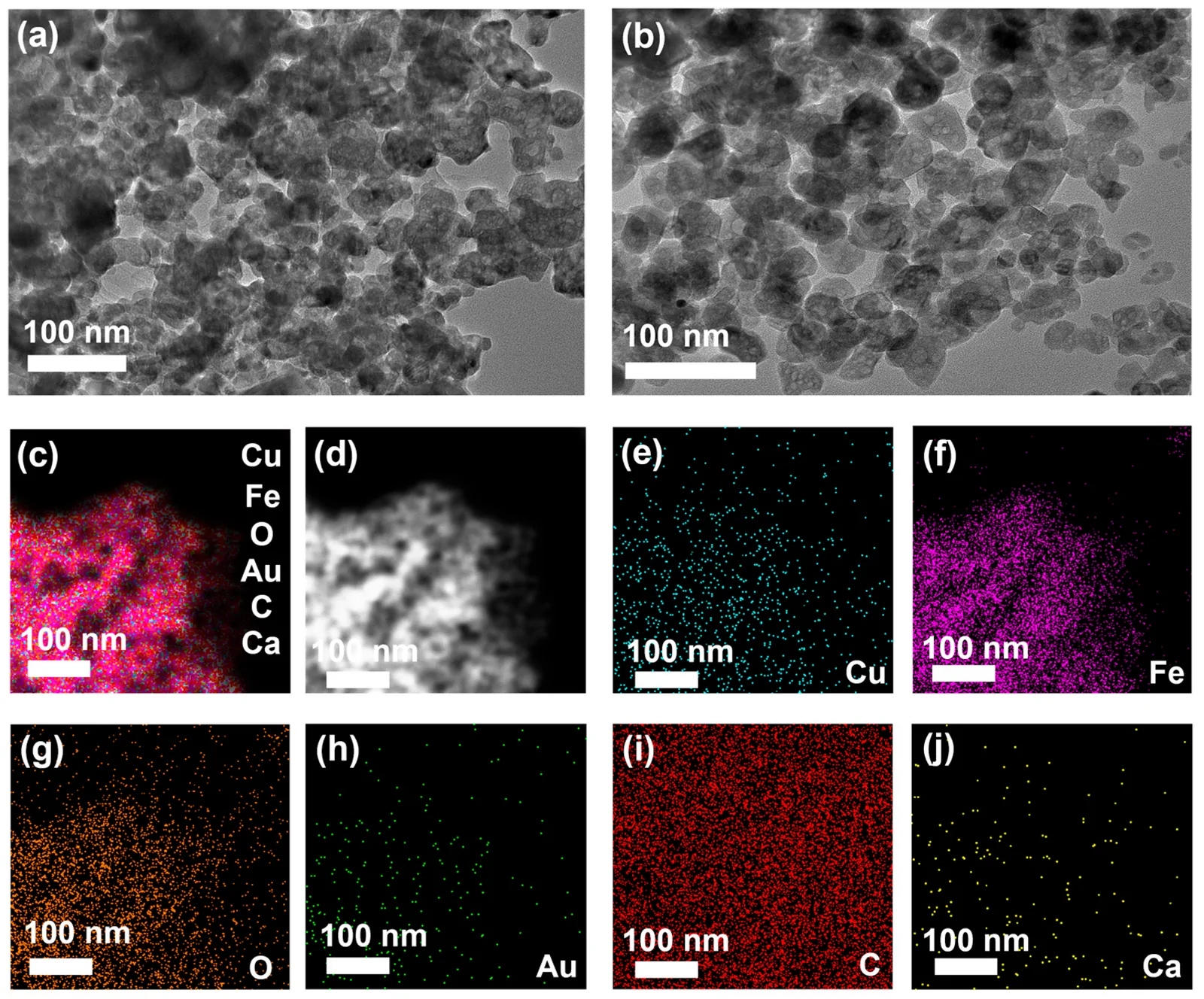
(**a**,**b**) TEM images and (**c**–**j**) elemental mapping of synthesized hydrogel.

The TEM images revealed nanoscale inorganic domains dispersed throughout the hydrogel matrix, with the ferrite nanoparticles exhibiting a predominantly quasi-spherical morphology (Figure 5a,b). The corresponding EDS elemental maps demonstrate the relatively uniform spatial distribution of Cu, Fe, Au, C, O, and Ca throughout the examined region (Figure 5c–j). The strong co-localization of Cu and Fe is consistent with the CuFe_2_O_4_ phase, while the distributed Au signal confirms the incorporation of Au species within the composite. The broad distribution of C and O is consistent with the alginate matrix, while the presence of Ca confirms its incorporation into the hydrogel network. The uniform elemental distribution and nanoscale integration of the Au and CuFe_2_O_4_ components support the formation of the hybrid nanozyme architecture.

**Figure 6:**
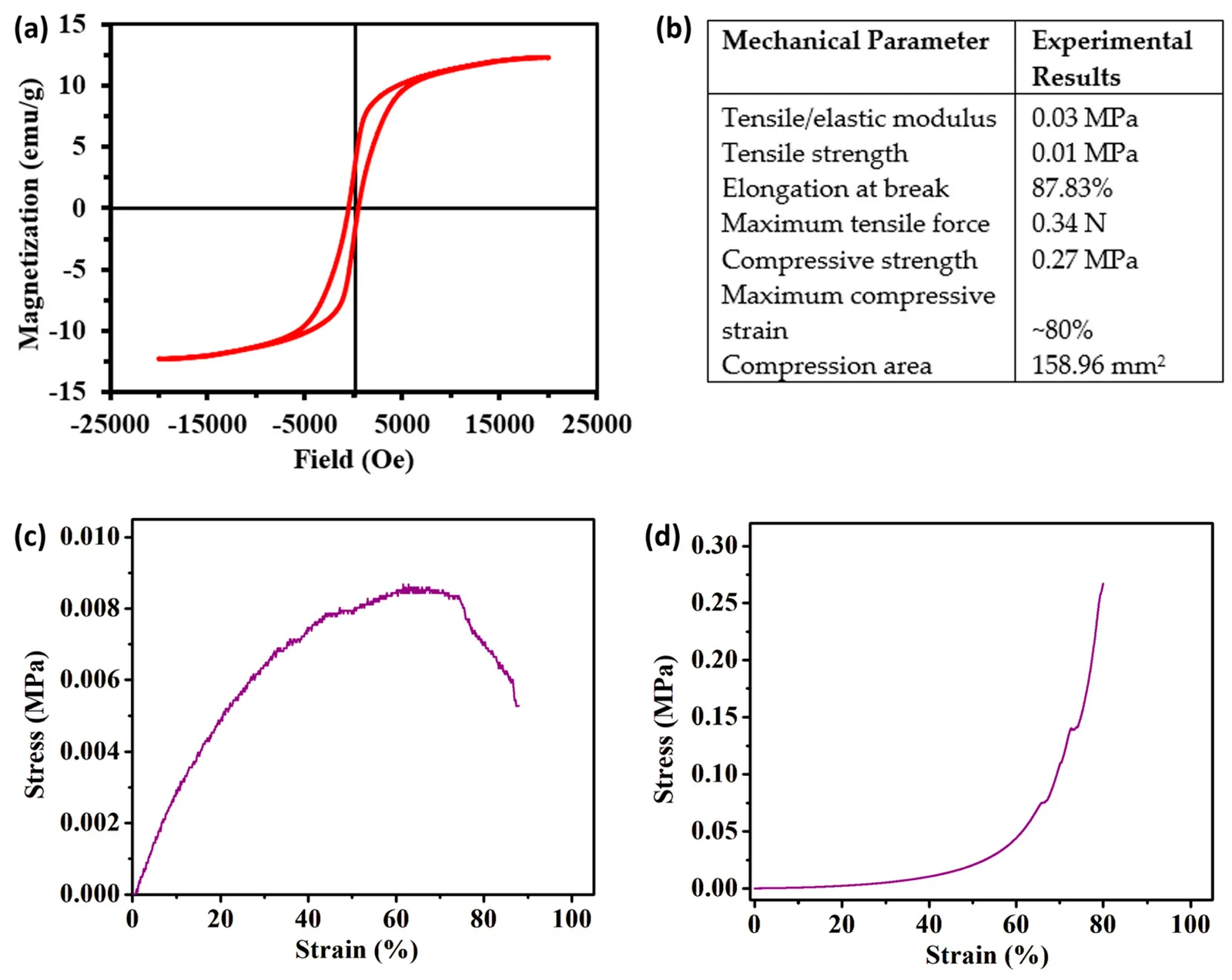
(**a**) Hysteresis loop; (**b**) rheological data; (**c**) tensile strength; (**d**) compressibility potential of hydrogel.

Magnetic Analysis: The magnetic properties of the Au-CuFe_2_O_4_/alginate hydrogel were investigated using vibrating-sample magnetometry (VSM) at room temperature over a magnetic field range of ±10 kOe. The hysteresis loop (Figure 6) exhibited ferromagnetic behaviour with a saturation magnetization (M_s_) of 12.35 emu g^−1^ and a remanent magnetization (M_r_) of 2.64 emu g^−1^.

In spinel ferrites, the saturation magnetization depends on the distribution of Fe^3+^ ions between the tetrahedral (A) and octahedral (B) sites, which is influenced by the site preference of the divalent metal cation (M^2+^). Cu^2+^ ions preferentially occupy octahedral sites, leading to strong antiferromagnetic coupling between Fe^3+^ ions at the A and B sites, which contributes to the observed high saturation magnetization [49]. The magnetic responsiveness of the hydrogel enables convenient magnetic handling and retrieval, while magnetically guided delivery and magnetically controlled release remain potential directions for future investigation.

Inductively Coupled Plasma–Optical Emission Spectroscopy (ICP-OES): To quantify the loading of nanoparticles within the hydrogel, ICP-OES analysis was performed on acid-digested samples. As shown in Table 2, the hydrogel contained 7.16 ± 0.01% Fe, 3.63 ± 0.01% Cu, and 0.38 ± 0.01% Au. The Fe–Cu molar ratio was calculated to be approximately 1.97:1, which closely matches the theoretical ratio of 2:1 for CuFe_2_O_4_, thereby confirming the successful synthesis of copper ferrite nanoparticles. The Au content of 0.38% confirms the efficient in situ formation and immobilization of gold nanoparticles within the hydrogel network. These results demonstrate the successful incorporation of both CuFe_2_O_4_ and Au nanoparticles in the alginate hydrogel matrix.

Mechanical Properties of the Au-CuFe_2_O_4_ Hydrogel: The mechanical properties of the Au-CuFe_2_O_4_/alginate hydrogel were evaluated under tensile and compressive loading. In the tensile test, the hydrogel exhibited an elastic modulus of approximately 0.03 MPa and an elongation at break of 87.83%, with a maximum tensile stress of approximately 0.01 MPa. The stress–strain profile showed a progressive increase in stress with increasing strain, followed by a decrease in stress at higher deformation, indicating failure of the hydrogel network. Under compression, the hydrogel displayed a highly nonlinear stress–strain response, with the stress increasing progressively with increasing compressive strain and reaching approximately 0.27 MPa at around 80% strain. The relatively high compressive strength demonstrates that the hydrogel can withstand substantial deformation without catastrophic failure, which is desirable for a flexible hydrogel intended for wound-dressing applications. The swelling ratio of the Au-CuFe_2_O_4_/alginate hydrogel was determined by gravimetric analysis. The dried hydrogel (1.34 g) swelled to 4.20 g after 24 h and 5.82 g after 48 h, corresponding to swelling ratios of 213.4% and 334.3%, respectively. The high water-uptake capacity confirms the hydrophilic nature of the alginate matrix and suggests its potential as an effective wound-dressing material.

### 2.3. Peroxidase (POD) and Glutathione (GSH)-like Activities

**Figure 7:**
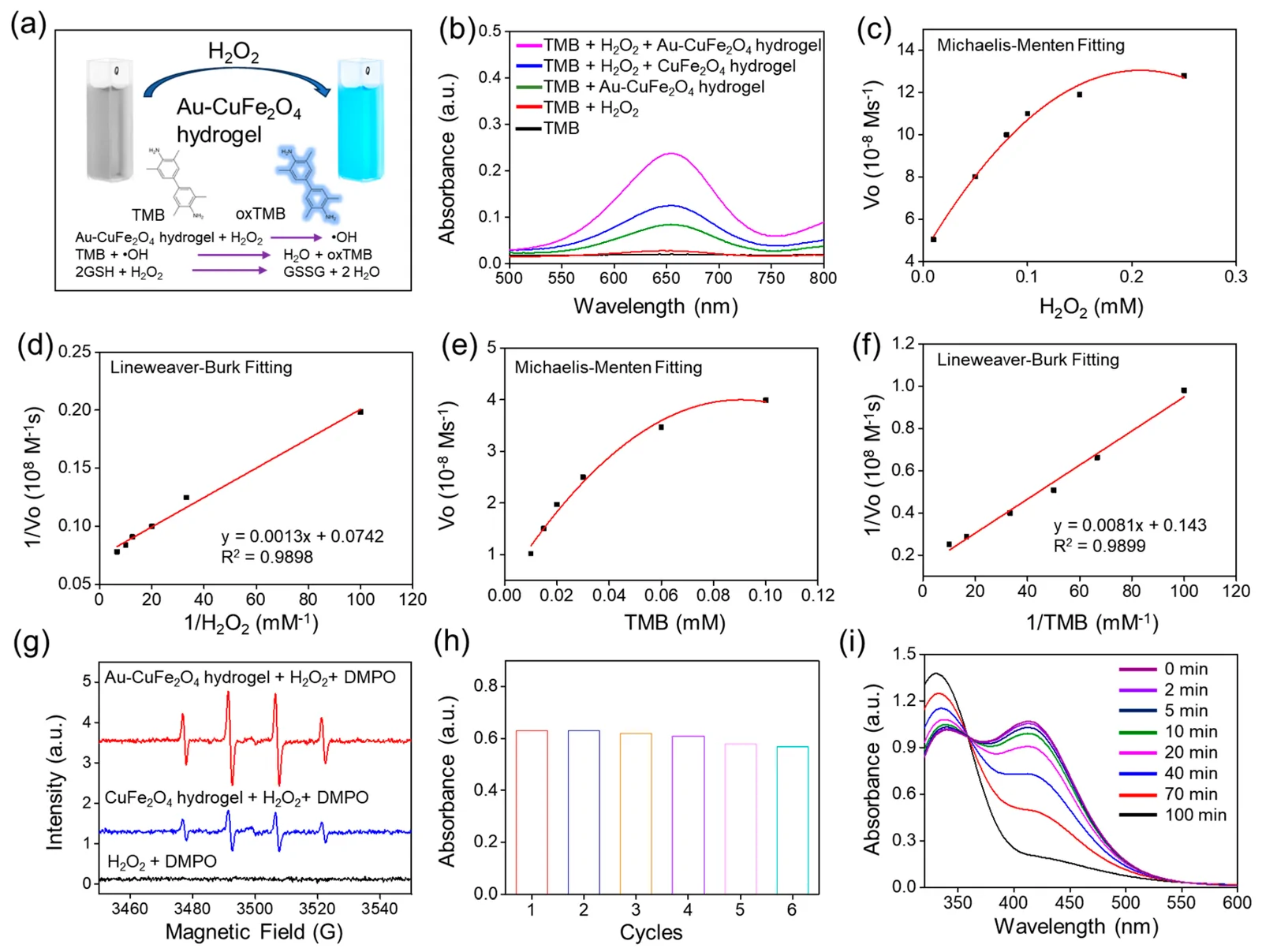
(**a**) Schematic illustration of the POD-like catalytic process of Au-CuFe_2_O_4_ hydrogel. (**b**) Visible absorption spectra of TMB solution treated with various formulations (TMB, TMB + H_2_O_2_, TMB + Au-CuFe_2_O_4_ hydrogel, TMB + H_2_O_2_ + CuFe_2_O_4_ hydrogel, and TMB + H_2_O_2_ + Au-CuFe_2_O_4_ hydrogel). (**c**) Michaelis–Menten curves at different concentrations of H_2_O_2_. (**d**) Double-reciprocal plots for determining the kinetic constants of Au-CuFe_2_O_4_ hydrogel for H_2_O_2_ substrate. (**e**) Michaelis–Menten curves at different concentrations of TMB. (**f**) Double-reciprocal plots for determining the kinetic constants of three nanocrystals for TMB substrate. (**g**) Detection of ^•^OH radicals using DMPO as a trapping agent by EPR spectra. (**h**) Recyclability graph of Au-CuFe_2_O_4_ hydrogel. (**i**) GSH-depleting ability of Au-CuFe_2_O_4_ hydrogel using DTNB as the trapping agent for sulfhydryl (-SH) groups.

The POD-like activity of the Au-CuFe_2_O_4_ nanozymes was first investigated. POD-mimicking enzymes catalyse the decomposition of H_2_O_2_ to generate ROS in acidic media. The generated ROS can further oxidize 3,3′,5,5′-tetramethylbenzidine (TMB) to produce blue-coloured oxidized TMB (oxTMB), which exhibits a characteristic absorption maximum at 652 nm (Figure 7a) [50]. TMB was employed as a chromogenic substrate to detect ROS generation (Figure 7b). In marked contrast to the other three control groups, a strong absorbance at 652 nm was observed upon the addition of both H_2_O_2_ and Au-CuFe_2_O_4_ hydrogel, confirming that the Au-CuFe_2_O_4_ hydrogel effectively catalyses H_2_O_2_ to generate ROS.

To further evaluate the catalytic efficiency, the steady-state kinetics of the Au-CuFe_2_O_4_ hydrogel was examined at varying H_2_O_2_ concentrations, and the corresponding Michaelis–Menten fitting curves and kinetic parameters were obtained. The analysis (Figure 7c,d) revealed that the Au-CuFe_2_O_4_ hydrogel exhibited a maximum reaction velocity (V_max_) of 13.47 × 10^−8^ M s^−1^ and a Michaelis–Menten constant (K_m_) of 0.01 mM, indicating a high affinity for the H_2_O_2_ substrate. Similarly, Michaelis–Menten kinetics was evaluated by varying the TMB concentration (Figure 7e), and the kinetic parameters were derived from the double-reciprocal Lineweaver–Burk plots (Figure 7f). The calculated K_m_ and V_max_ values for TMB were 0.05 mM and 6.99 × 10^−8^ M s^−1^, respectively (Table 3). The relatively low Km and high V_max_ values suggest a strong substrate affinity and a high catalytic turnover rate, providing a promising foundation for antibacterial applications.

On this basis, we performed electron paramagnetic resonance (EPR) tests for CuFe_2_O_4_ hydrogel and Au-CuFe_2_O_4_ hydrogel to further explore the ROS potential. The EPR spectra showed that the signal arising due to DMPO/^•^OH was negligible in the absence of hydrogels. In contrast, when the Au-CuFe_2_O_4_ hydrogel was added in the presence of H_2_O_2_, a strong EPR spectrum with a 1:2:2:1 line appeared as compared to the CuFe_2_O_4_ hydrogel (Figure 7g). Consequently, the results indicate that the prepared Au-CuFe_2_O_4_ hydrogel can generate ^•^OH during the decomposition of H_2_O_2_. To check the recyclability of the hydrogel, the hydrogel was removed from the cuvette solution using an external magnet and then washed with DI water and dried. It could be reused for up to six cycles, and no significant differences were observed in the absorbance of the sample (Figure 7h).

Additionally, the glutathione (GSH) consumption capacity of the Au-CuFe_2_O_4_ hydrogel was evaluated (Figure 7i). The material exhibited a significant GSH-depleting ability, further highlighting its potential for disrupting the antioxidant defence system of bacteria.

To benchmark the catalytic performance of the Au-CuFe_2_O_4_ hydrogel, its kinetic parameters were compared with those of natural horseradish peroxidase (HRP) and previously reported nanozymes (Table 4). The Au-CuFe_2_O_4_ hydrogel exhibited a remarkably low K_m_ value for H_2_O_2_, as compared to HRP and other nanozymes. This indicates a superior substrate affinity of the Au-CuFe_2_O_4_ hydrogel toward H_2_O_2_. Furthermore, the V_max_ value is comparable to or higher than that of most reported nanozymes, demonstrating a high catalytic efficiency of the Au-CuFe_2_O_4_ hydrogel.

**Figure 8:**
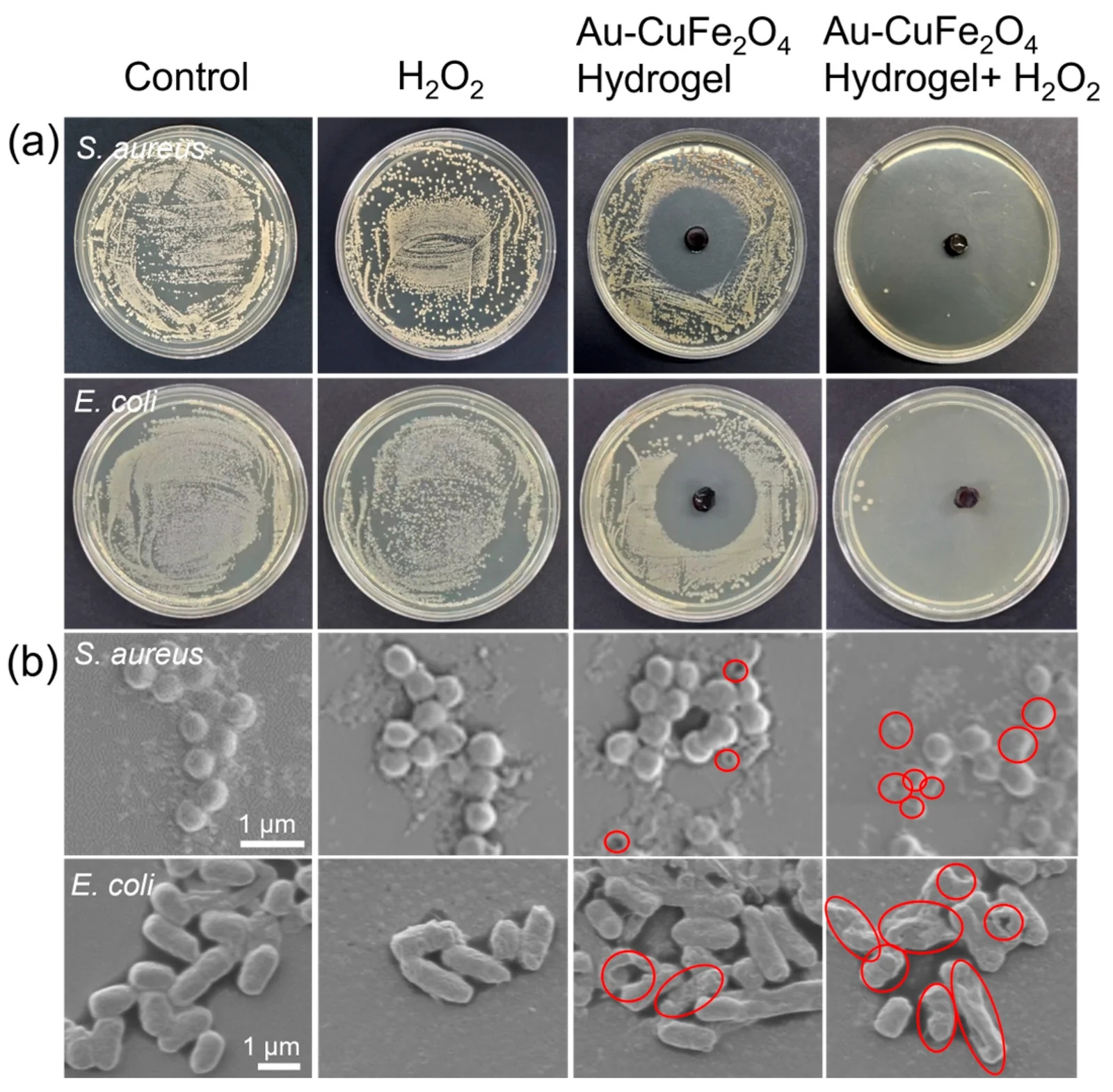
Comparative antibacterial studies. (**a**) The plate samples showing colonies of *S. aureus* and *E. coli.* (**b**) SEM images of *S. aureus* and *E. coli* after various treatments.

Hydrogen peroxide is widely used to treat bacterial infections. However, relative to H_2_O_2_, the hydroxyl radical (^•^OH) is significantly more reactive and can cause serious oxidative damage to lipids and amino acids [51,52]. Therefore, the Au-CuFe_2_O_4_ hydrogel was expected to enhance the bacterial killing efficiency of H_2_O_2_. Based on this hypothesis, we assessed the antibacterial efficacy of H_2_O_2_ both in the absence and presence of Au-CuFe_2_O_4_ hydrogel, quantifying the colony-forming units (CFU) on agar plates against Gram-positive *Staphylococcus aureus* (*S. aureus*) and Gram-negative *Escherichia coli* (*E. coli*). The plate cultures of *E. coli* and *S. aureus* revealed that bacterial growth was significantly inhibited in the presence of Au-CuFe_2_O_4_ hydrogel combined with H_2_O_2_ (10 mM). In contrast, the groups treated with H_2_O_2_ alone or Au-CuFe_2_O_4_ hydrogel alone exhibited negligible antibacterial activity (Figure 8a). Evidently, the combination of H_2_O_2_ and Au-CuFe_2_O_4_ hydrogel produced enhanced antibacterial activity compared with either treatment alone.

Scanning electron microscopy (SEM) was employed to observe the morphological changes in *S. aureus* and *E. coli* cells following treatment. Bacterial cells treated with Au-CuFe_2_O_4_ hydrogel (10 mg) in the presence of H_2_O_2_ (10 mM) exhibited severe morphological and structural damage, including surface folds, ruptures, and loss of cellular integrity (Figure 8b). In contrast, cells incubated with H_2_O_2_ alone showed negligible morphological alterations. These results confirm that Au-CuFe_2_O_4_ nanozymes possess significant POD-like activity, effectively killing bacteria through oxidative damage.

**Figure 9:**
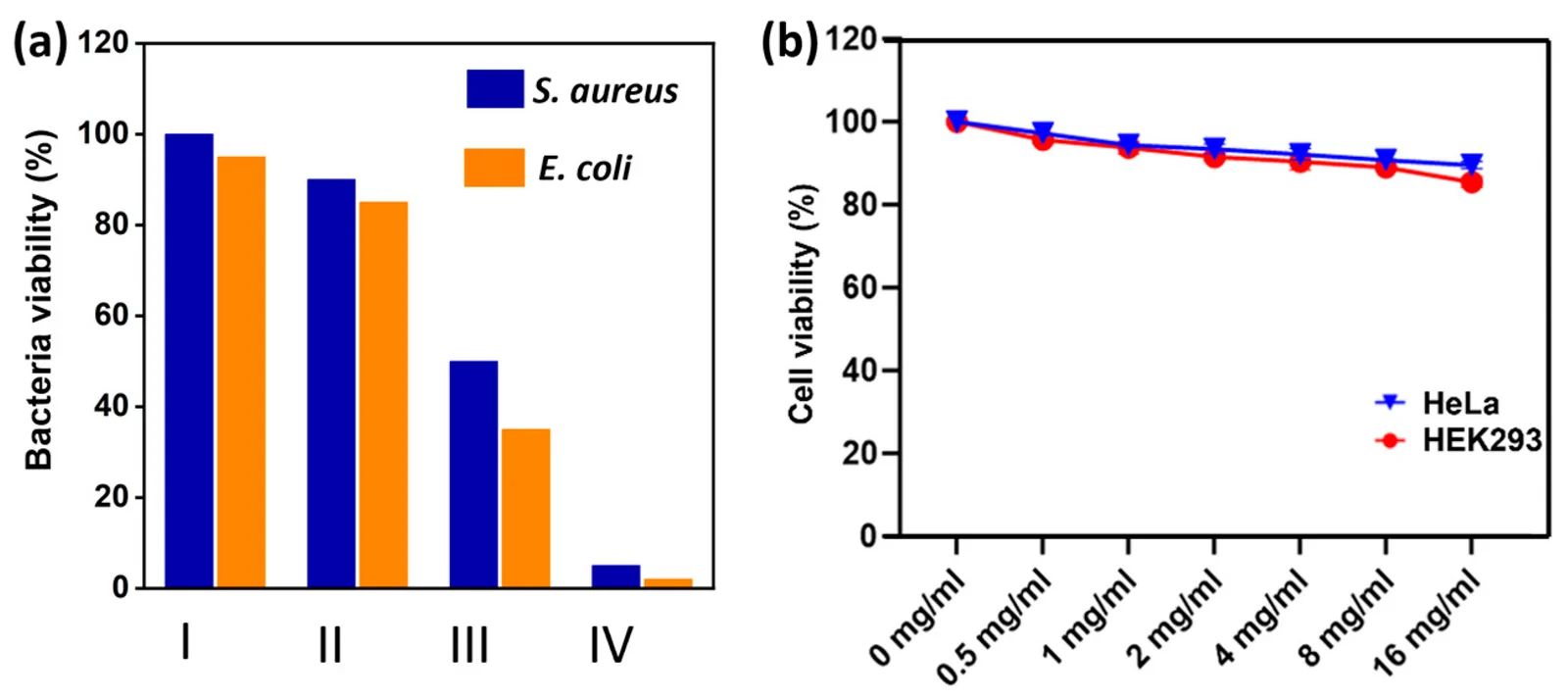
(**a**) Viability percentage of cultured bacterial cells treated with (I) NaAc-HAc buffer (0.2 M, pH = 4) as control, (II) H_2_O_2_, (III) hydrogel, and (IV) H_2_O_2_ in the presence of hydrogel. (**b**) In vitro biosafety evaluation of Au-CuFe_2_O_4_ hydrogel. HeLa tumour cells and normal HEK293 cells were treated with Au-CuFe_2_O_4_ hydrogel at serial concentrations, followed by cell viability measurement.

Cell Viability Assay: HeLa cells and normal HEK293 cells were incubated with Au-CuFe_2_O_4_ hydrogel at gradient concentrations ranging from 0 to 16 mg mL^−1^ to evaluate the in vitro biosafety of the material (Figure 9). The cell viabilities of both HeLa and HEK293 cells exhibited a slight declining trend with the increase in material concentration. Even at the maximum tested concentration (16 mg mL^−1^), the viabilities of the two cell lines remained above 85%, without obvious cytotoxicity. Collectively, these results demonstrate favourable in vitro cytocompatibility of the Au-CuFe_2_O_4_ hydrogel within the tested concentration range of 0–16 mg mL^−1^, supporting its further investigation for potential biomedical applications.

The present findings establish the Au-CuFe_2_O_4_ hydrogel as a promising antibacterial nanozyme platform with favourable in vitro cytocompatibility, swelling, mechanical, and magnetic properties. While the observed >85% cell viability supports its cytocompatibility, further evaluation of haemocompatibility and in vivo wound-healing efficacy is required to establish its biomedical potential. Similarly, antibacterial activity against clinically isolated or multidrug-resistant strains, quantitative Cu and Fe ion-release kinetics, and hydrolytic/enzymatic degradation under physiological and wound-like conditions warrant further investigation. Although enhanced antibacterial activity was observed in the presence of H_2_O_2_, the effect is described as an enhanced antibacterial response. Additional component-specific controls would further clarify the individual contributions of the hydrogel constituents. Finally, the demonstrated magnetic responsiveness and reusability support magnetic handling and retrieval, while magnetically guided delivery and controlled release remain potential future applications. These considerations notwithstanding, the combination of antibacterial activity and favourable physicochemical and in vitro cytocompatibility characteristics provides a promising basis for future investigation of the hydrogel as a wound-dressing material.

## 3. Conclusions

We successfully developed a gold-dispersed CuFe_2_O_4_/alginate magnetic hydrogel with potent POD-like activity and enhanced antibacterial properties. The hydrogel effectively generates ROS, depletes glutathione, and causes oxidative damage to bacterial cells, achieving significantly improved antibacterial efficacy against both *S. aureus* and *E. coli* at reduced H_2_O_2_ concentrations. Cytocompatibility studies confirmed favourable in vitro biocompatibility (>85% cell viability), and the hydrogel displayed good mechanical properties and reusability. The incorporation of Au nanoparticles substantially enhanced the catalytic performance compared to the CuFe_2_O_4_ hydrogel without Au. This nanozyme-integrated hydrogel represents a promising candidate for further investigation in antibacterial wound-dressing applications, owing to its magnetic responsiveness, favourable in vitro cytocompatibility, and enhanced antibacterial activity.

## 4. Materials and Methods

Materials: Ferric nitrate (Fe(NO_3_)_3_·9H_2_O), copper nitrate (Cu(NO_3_)_2_·3H_2_O), and HAuCl_4_·3H_2_O were obtained from Merck (Shanghai, China). Hydrogen peroxide (H_2_O_2_, 30%) was purchased from Shanghai Aladdin Bio-Chem Technology Co., Ltd. 5,5-Dithiobis (2-nitrobenzoic acid) (DTNB) and glutathione (GSH) were obtained from Shanghai Aladdin Bio-Chem Technology Co., Ltd. (Shanghai, China). PBS (pH 7.4, 10 mM) was purchased from Gibco Life Technologies (Shanghai, China). Ethanol was obtained from Shanghai Macklin Biochemical Co., Ltd. (Shanghai, China). *E. coli* was obtained from the College of Life Sciences, Nanjing University. *S. aureus* was obtained from the Shenyang Institute of Applied Ecology, Chinese Academy of Sciences. Ultrapure water (18.2 MΩ) was used throughout the experiments. We used Cell Counting Kit-8 (Cat. No. CP736, DOJINDO Laboratories, Kumamoto, Japan), a clean bench (Model SW-CJ-1FD, Suzhou Purification Equipment, Suzhou, China), and a microplate reader (Model ELx800, BioTek, Winooski, VT, USA).

Instruments: Scanning electron microscope (SEM) images and energy-dispersive X-ray spectroscopy (EDS) data were obtained on a field-emission scanning electron microscope (Supra 55, Carl Zeiss Microscopy GmbH, Jena, Germany). Transmission electron microscopy (TEM) images were obtained on a JEM-2100PULS (JEOL Ltd., Tokyo, Japan). Inductively coupled plasma–optical emission spectroscopy (ICP-OES) was recorded on an Agilent 5110 (Melbourne, Australia). Fourier-transform infrared (FT-IR) spectra were recorded using a Bruker Alpha II spectrometer (Bruker Optics, Ettlingen, Germany). Sonication was done by using the PowerSonic405 bath sonicator (Hwashin Tech Co., Ltd., Seoul, Republic of Korea). The vibrating-sample magnetometer (VSM) was recorded using VSM3105, East Changing Technologies, Inc., Beijing, China. The UV–visible absorption spectra were recorded using a JASCO V-750 spectrophotometer (Tokyo, Japan). Mechanical properties were measured using CMT4204 (Shenzhen Meister Co., Ltd., Shenzhen, China).

Synthesis of CuFe_2_O_4_ *via* Hydrothermal Approach: In this method, to prepare nanoferrites, stoichiometric amounts of metal salt and ferric nitrate were dissolved in a minimal amount of distilled water. The pH of the solution was adjusted to 7.5 with the addition of ammonia solution. The volume of the solution was made up to 120 mL by the addition of distilled water followed by continuous stirring for 2 h. The solution was then transferred to a Teflon autoclave and treated hydrothermally at 160 °C for 15 h [62,63]. The precipitates were cooled to room temperature and were thoroughly washed with distilled water and acetone. Finally, the obtained precipitates were dried in an oven at 60 °C for 10 h.

Synthesis of Hydrogel: A 100 mM CaCl_2_ solution was prepared by dissolving CaCl_2_·2H_2_O in deionized (DI) water. Sodium alginate (1.0 g) was dissolved in 40 mL of DI water at 35–40 °C under continuous stirring to obtain a homogeneous solution. CuFe_2_O_4_ nanoparticles (200 mg) were dispersed in DI water containing gelatine (10–20 mg) as a stabilizer and added dropwise to the alginate solution under stirring. Subsequently, an aqueous HAuCl_4_ solution (0.010 g in 3–4 mL DI water) was introduced slowly into the mixture. The resulting suspension was transferred into the CaCl_2_ solution to induce ionic crosslinking and form the hydrogel. The obtained hydrogel was immersed in 0.01 M ascorbic acid solution for 1–2 h at room temperature to reduce Au^3+^ ions into in situ-formed Au nanoparticles. Finally, the hydrogel was thoroughly washed with DI water and stored for further characterization.

The POD activity of the Au-CuFe_2_O_4_ hydrogel was assessed by tracking TMB oxidation in the presence of H_2_O_2_. First, 10 mg of Au-CuFe_2_O_4_ hydrogel was added to 200 μL of 6 mM TMB and 12 mM H_2_O_2_ in DI water. The total volume was then brought to 3 mL with buffer solution. The characteristic absorption peak at 652 nm was monitored over time using a UV–vis spectrophotometer. Kinetic experiments were performed at fixed TMB concentrations (200 μL) and Au-CuFe_2_O_4_ hydrogel amounts (10 mg) while varying H_2_O_2_ concentrations (0.01 mM, 0.05 mM, 1 mM, 1.5 mM, and 2.5 mM). Kinetic experiments were performed at fixed H_2_O_2_ concentrations (100 μL) and Au-CuFe_2_O_4_ hydrogel amounts (10 mg) while varying the TMB concentrations (0.01 mM, 0.015 mM, 0.02 mM, 0.03 mM, 0.06 mM and 0.1 mM). The catalytic kinetics were fitted using the Michaelis–Menten equation:

$$v_{o}=\frac{\Delta A}{kb\Delta t}$$

and the Lineweaver–Burk equation was employed to obtain the kinetic parameters:

$$v_{o}=\frac{k_{m}}{v_{max}}\cdot \frac{1}{\left[S\right]}+\frac{1}{v_{max}}$$

where *v_o_* is the initial velocity (M s^−1^), *v_max_* is the maximum reaction velocity (M s^−1^), *k_m_* is the Michaelis constant (mM), *∆A* represents the change in absorbance at 652 nm over the selected time interval *∆t* (180 s), and *[S]* is the concentration of the substrate (mM). All of the tests were performed three times to improve the statistics.

Glutathione Depletion Activity: To validate glutathione depletion ability, Au-CuFe_2_O_4_ hydrogel (10 mg) was added to a PBS solution containing glutathione (10 mM). The mixed solutions were shaken and incubated for different times (0 min to 100 min). Then, 100 μL DTNB solutions (5 mM) were added to the mixture. Finally, the absorbance spectra were measured to observe the glutathione depletion level.

Bacterial Culture and Antibacterial Activity: Gram-positive *S. aureus* and Gram-negative *E. coli* were cultured overnight in lysogeny broth (LB) medium under shaking conditions. *S. aureus* was grown at 30 °C with agitation at 180 rpm, while *E. coli* was grown at 37 °C with agitation at 160 rpm. Once the optical density of the bacterial suspension reached approximately 1.5 (measured at absorbance), the cultures were diluted 10-fold with fresh LB medium. Then, 100 μL of the diluted bacterial suspension was transferred into a 2 mL centrifuge tube. The bacteria were harvested by centrifugation and resuspended in phosphate-buffered saline (PBS, 0.2 M, pH 7.2).

Subsequently, 800 μL of buffer and 100 μL of H_2_O_2_ (10 mM) were added to the centrifuged bacterial pellet. The same procedure was repeated for three additional groups, with the following modifications: buffer only, H_2_O_2_ only, and hydrogel only, while all other conditions remained unchanged. For the hydrogel treatment, 10 mg of Au-CuFe_2_O_4_ hydrogel was placed directly onto the culture plates.

The four mixtures were incubated for 2 h under specific conditions: *E. coli* samples were shaken at 160 rpm and 37 °C, while *S. aureus* samples were shaken at 180 rpm and 30 °C. Following incubation, 150 μL of each mixture was evenly spread onto solid agar medium, and an additional 10 mg of Au-CuFe_2_O_4_ hydrogel was placed directly onto the plates. Bacterial growth was assessed after further incubation at the specified temperatures for 24 h. The remaining sample solutions were collected and prepared for scanning electron microscopy (SEM) to analyse bacterial morphology.

Cell Viability Test: Cells were digested and counted, and a cell suspension was adjusted to a concentration of 5 × 10^4^ cells mL^−1^. A total of 100 μL of the cell suspension was added to each well of a 96-well cell culture plate. The plate was incubated at 37 °C in a 5% CO_2_ incubator for 24 h. Subsequently, corresponding hydrogels were added to designated groups, and a negative control group was set up. After another 24 h of incubation, 5 μL of CCK-8 reagent was added to each well. The OD value was measured at 450 nm, and the inhibition rate was calculated.

## Figures and Tables

**Table 1:** Lattice parameter a (Å) and crystallite size D_hkl_ (nm) of the sample prepared.

Method of Preparation	Ferrites	A (Å)	D_hkl_ ± 2 (nm)
Hydrothermal	CuFe_2_O_4_	8.42	26

**Table 2:** Elemental composition and stoichiometric analysis of the Au-CuFe_2_O_4_/alginate hydrogel determined by ICP-OES.

Element	C_0_ (mg L^−1^) ^a^	Dilution (f)	C_1_ (mg L^−1^) ^b^	Content (mg kg^−1^)	Content (%)
Fe	102.65 ± 0.09	1	102.65 ± 0.09	71,633.52 ± 65.59	7.16 ± 0.01
Cu	5.20 ± 0.01	10	52.04 ± 0.08	36,312.86 ± 55.87	3.63 ± 0.01
Au	5.46 ± 0.01	1	5.46 ± 0.01	3811.77 ± 9.20	0.38 ± 0.01

(a) Concentration in the measured test solution. (b) Concentration in the original sample solution after accounting for dilution.

**Table 3:** Kinetic parameters of Au-CuFe_2_O_4_ toward the oxidation of TMB by H_2_O_2_.

Catalyst	Substrate	K_m_ (mM)	V_max_ (10^−8^ M s^−1^)
Au-CuFe_2_O_4_ hydrogel	H_2_O_2_	0.01	13.47
	TMB	0.05	6.99

**Table 4:** Comparison of the kinetic parameters of the Au-CuFe_2_O_4_ hydrogel with previously reported nanozymes.

Material	K_m_ (mM) for H_2_O_2_	V_max_ (10^−8^ M s^−1^)	Reference
Au-CuFe_2_O_4_	0.01	13.47	This work
Fe_3_O_4_	154	9.78	[13]
CuFe_2_O_4_	0.31	3.81	[20]
PtNPs	5.42	15.53	[51]
HRP	3.70	8.71	[52]
Cu_2_[Fe(CN)_6_]	14.44	0.745	[53]
Cl-FeNCDs	0.2142	1.048	[54]
FcP NPs@PDA	2.36	0.726	[55]
MgCaFe-LDH@Au NSs	29.698	7.19	[56]
Hemin-BTO	72.96	0.195	[57]
Cu_2_O/Au–Pt@MOF	22.4	0.405	[58]
a-Gd(OH)_3_	208.39	4.54	[59]
Au-Cu_4_O_3_/re-LDHs	17.44	1.79	[60]
Fe^3+^/Cu^2+^ dis-SAzyme	34.8	0.36	[61]

## Data Availability

The original contributions presented in this study are included in the article. Further inquiries can be directed to the corresponding authors.
